# A small number of early introductions seeded widespread transmission of SARS-CoV-2 in Québec, Canada

**DOI:** 10.1186/s13073-021-00986-9

**Published:** 2021-10-28

**Authors:** Carmen Lía Murall, Eric Fournier, Jose Hector Galvez, Arnaud N’Guessan, Sarah J. Reiling, Pierre-Olivier Quirion, Sana Naderi, Anne-Marie Roy, Shu-Huang Chen, Paul Stretenowich, Mathieu Bourgey, David Bujold, Romain Gregoire, Pierre Lepage, Janick St-Cyr, Patrick Willet, Réjean Dion, Hugues Charest, Mark Lathrop, Michel Roger, Guillaume Bourque, Jiannis Ragoussis, B. Jesse Shapiro, Sandrine Moreira

**Affiliations:** 1 McGill Genome Centre, Montreal, QC Canada; 2 Department of Microbiology and Immunology, McGill University, Montreal, QC Canada; 3 Département de Sciences Biologiques, Université de Montréal, Montreal, QC Canada; 4 Laboratoire de Santé Publique du Québec, Institut National de Santé Publique, Montreal, QC Canada; 5 Canadian Center for Computational Genomics, Montreal, QC Canada; 6 Department of Human Genetics, McGill University, Montreal, QC Canada; 7 Calcul Québec, Montreal, QC Canada; 8 Ecole de santé publique, Université de Montréal, Montreal, QC Canada; 9 Département de Microbiologie, infectiologie et Immunologie, Université de Montréal, Montreal, QC Canada; 10 Department of Bioengineering, McGill University, Montreal, QC Canada

## Abstract

**Background:**

Québec was the Canadian province most impacted by COVID-19, with 401,462 cases as of September 24th, 2021, and 11,347 deaths due mostly to a very severe first pandemic wave. In April 2020, we assembled the Coronavirus Sequencing in Québec (CoVSeQ) consortium to sequence SARS-CoV-2 genomes in Québec to track viral introduction events and transmission within the province.

**Methods:**

Using genomic epidemiology, we investigated the arrival of SARS-CoV-2 to Québec. We report 2921 high-quality SARS-CoV-2 genomes in the context of > 12,000 publicly available genomes sampled globally over the first pandemic wave (up to June 1st, 2020). By combining phylogenetic and phylodynamic analyses with epidemiological data, we quantify the number of introduction events into Québec, identify their origins, and characterize the spatiotemporal spread of the virus.

**Results:**

Conservatively, we estimated approximately 600 independent introduction events, the majority of which happened from spring break until 2 weeks after the Canadian border closed for non-essential travel. Subsequent mass repatriations did not generate large transmission lineages (> 50 sequenced cases), likely due to mandatory quarantine measures in place at the time. Consistent with common spring break and “snowbird” destinations, most of the introductions were inferred to have originated from Europe via the Americas. Once introduced into Québec, viral lineage sizes were overdispersed, with a few lineages giving rise to most infections. Consistent with founder effects, the earliest lineages to arrive tended to spread most successfully. Fewer than 100 viral introductions arrived during spring break, of which 7–12 led to the largest transmission lineages of the first wave (accounting for 52–75% of all sequenced infections). These successful transmission lineages dispersed widely across the province. Transmission lineage size was greatly reduced after March 11th, when a quarantine order for returning travellers was enacted. While this suggests the effectiveness of early public health measures, the biggest transmission lineages had already been ignited prior to this order.

**Conclusions:**

Combined, our results reinforce how, in the absence of tight travel restrictions or quarantine measures, fewer than 100 viral introductions in a week can ensure the establishment of extended transmission chains.

**Supplementary Information:**

The online version contains supplementary material available at 10.1186/s13073-021-00986-9.

## Background

Over a year into the SARS-CoV-2 pandemic, whole-genome sequencing combined with phylogenetic analysis has emerged as an essential tool to track the local and global spread and evolution of the virus [1, 2]. While the pandemic is global by definition, regional instances of viral introductions and spread provide “natural experiments” to gain insights into general patterns. For example, Russia, Scotland, Zimbabwe, and Massachusetts all experienced dozens to a few hundred independent introduction events of the virus from different locations [3–6]. A phylogenetic analysis of Massachusetts in particular found that most introduced viruses went extinct, while a minority of introductions were highly successful, consistent with superspreading dynamics [5]. Phylogenetic analysis can also identify cryptic transmission chains unidentified by contact tracing or travel history [7, 8]. Combinations of genomics, travel history, and contact tracing can provide deeper and more robust insights into transmission chains [9]. More recently, evidence has accumulated that transmissibility can be increased by adaptive mutations in the viral genome, such as amino acid change D614G in the spike protein [10, 11], or combinations of mutations, as in the B.1.1.7 Pangolin (PANGO) lineage [12], i.e., Alpha (WHO notation), that emerged in Southeast England in September 2020 and quickly became the predominant PANGO lineage in the UK [13]. The interplay between adaptive evolution, such as beneficial mutations, and stochastic factors, such as founder effects and superspreading, remains to be fully explored, and additional case studies are instructive to distinguish region-specific from generalizable features of the pandemic.

**Fig. 1:**
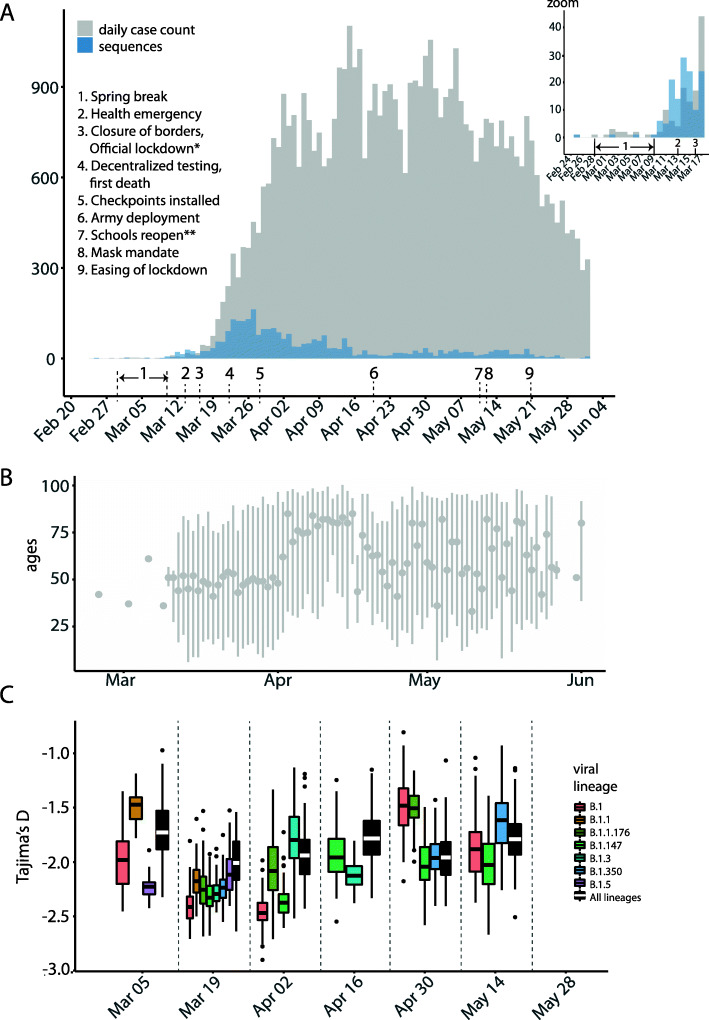
Timeline of COVID-19 cases and sequencing in Québec up to June 1. **A** Comparison of confirmed cases (grey) reported by public health authorities and high-quality sequences used in this study (blue) distributed by collection date. *Official lockdown included stay home orders and closure of schools and daycares. **Except schools in the city of Montreal. Timeline and control measures are from [15, 16]. Sample dates of sequences can be slightly offset from official daily case count due to reporting lags. **B** Age distribution of sequenced cases (mean and range shown). **C** Variation in viral epidemiological dynamics as estimated by Tajima’s *D*. Boxplots represent 99 resampled estimates of Tajima’s *D* from random resamplings of 20 genome sequences for each 2-week time period. Tajima’s *D* values are only estimated for PANGO lineages with at least 20 sequences in a given time period

The province of Québec (QC) was the epicenter of the first wave in Canada of the SARS-CoV-2 pandemic (defined here up to June 1st, 2020). It is the second most populous province, with about half of its 8.5 million inhabitants in the densely populated Montréal metropolitan area. By June 1st, 2020, 5210 people in Québec had died of Coronavirus disease 2019 (COVID-19), of whom 70% were residents of long-term care facilities. When the first cases were reported in China and Europe, the Public Health Laboratory of Québec (LSPQ) developed a qPCR diagnostic test targeting SARS-CoV-2 E and N genes [14]. The first case of COVID-19 in Québec was detected on February 25th, 2020. Shortly after, Québec was the first large Canadian province to start its spring school holiday (“spring break;” February 29th to March 9th, 2020; Fig. 1A). It is believed that international travellers returning from spring break had a large impact on the epidemic [17]. The number of cases increased exponentially during March 2020 (Fig. 1A [18];). On March 13th, a public health emergency was declared, with schools, daycares, and most other public spaces closed on March 16th (“lockdown”). The closure of the Canadian border to non-essential travel was also announced March 16th and officially closed the night of the 17th, except for returning Canadian citizens who continued to enter the country after repatriation calls from the government. On March 20th, Québec reached the threshold of 100 cases per day and by March 28th random road checks were set up to discourage movement between regions within Québec and between neighboring provinces (i.e., movement between Gatineau, Québec, bordering Ottawa, Ontario, was restricted). In April 2020, the virus spread significantly in long-term care facilities overwhelming many of them, thus requiring redeployment of health care workers and by April 20th the Canadian Armed Forces sent personnel to the Montréal region to help. Having flattened the epidemic curve and with cases declining, public health measures began easing mid-May (Fig. 1A). A year and a half later, as of September 24th, 2021, Québec had suffered the highest death toll in Canada (over 11,000 dead) and among the highest death rates in the world (~ 132 deaths per 100,000).

In April 2020, we assembled the Coronavirus Sequencing in Québec (CoVSeQ) consortium of academic and government scientists (https://covseq.ca/) to sequence SARS-CoV-2 genomes in Québec. The CoVSeQ consortium is part of the Canadian COVID Genomic Network (CanCOGeN), a pan-Canadian cross-agency network for large-scale SARS-CoV-2 and human host sequencing (https://www.genomecanada.ca/en/cancogen). To better understand the early introductions and spread of SARS-CoV-2 in Québec during the first wave, we sequenced and analyzed 2921 high-quality consensus genome sequences obtained between mid-February and June 1st, 2020. We studied how these Québec sequences were related to 12,801 genomes sampled from elsewhere in Canada and internationally. We inferred geographical origins of introduction events by comparing travel history data with phylogenetic inference (Additional file 1: Figure S1) and estimated their likely arrival dates and subsequent spread. We conservatively estimated ~ 600 independent introduction events, mainly involving PANGO lineages of European origin, of which the most successful arrived early. Most viral lineages rapidly went extinct after being introduced into Québec, and only 7–12 introduction events gave rise to 50 or more sequenced cases. This overdispersed distribution of transmission lineage sizes was also documented in the greater Boston area and is thought to be driven by superspreading dynamics [5]. Consistent with founder effects, earlier introductions tended to give rise to more subsequent infections. Later introduction events were less successful, which also points to the effectiveness of public health measures in curbing local transmission.

## Methods

### Sampling and sequencing

COVID-19-positive cases were selected from all nasopharyngeal swabs sent to the Public Health Laboratory of Québec (Laboratoire de Santé Public du Québec, LSPQ) from the beginning of the pandemic until June 1st, 2020. In this period, we aimed to sequence as many samples as possible, though coverage dropped after late March 2020. Here we present 2921 high-quality SARS-CoV-2 genomes (Additional file 2: Table S1). Our sequencing strategy during this time had no specific bias towards outbreaks. The dataset is therefore relatively unbiased, with the caveat that the sampling strategy during the first wave was biased towards travellers, those with symptoms, and those directly exposed to a case. We attempted sequencing of all randomly selected qPCR-positive samples, without any filter for a particular cycle threshold (Ct) value. To protect patient confidentiality, in the publicly released data the first accurate date of sampling is set to March 10th, 2020. All samples taken before that are set to March 1st, and their real sampling dates are between February 25th and March 9th, 2020. The true sampling dates were used in phylogenetic analyses described below. Note that our dataset of 2921 consensus sequences is available through GISAID and the Canadian SARS-CoV-2 repository, CanCoGen’s VirusSeq Portal https://virusseq-dataportal.ca/explorer (Additional file 2: Table S1).

Total nucleic acid extraction was performed with the NucliSENS EASYMAG automated platform on 200 μL of nasopharyngeal swabs. The presence of SARS-CoV-2 was assessed by a qPCR diagnostic test targeting genes E and N [14]. Targeted SARS-CoV-2 amplification, library preparation, and sequencing were performed at the McGill Genome Centre as follows. Briefly, RNA samples were processed in a 96-well plate format, including positive and negative controls on each plate. A targeted amplification strategy was used based on the ARTIC V3 primer scheme (https://github.com/artic-network/artic-ncov2019 [19]) using the V3 primers only without adding the redundant V1 primers. For primer pairs 5, 17, 23, 26, 66, 70, 74, 91, 97, and 64, for which a lower coverage was observed, a separate additional low amplification (LA1) pool was prepared to increase the number of reads in the corresponding region. For post-PCR cleanup, pools 1 and 2 were combined, while pool LA1 was cleaned up separately, quantified, and added to the combined pools 1 + 2 in equimolar concentration. Samples from plates 1–4 were prepared for Nanopore sequencing as described: https://www.protocols.io/view/sars-cov-2-mcgill-nanopore-sequencing-protocol-sup-bjajkicn. For Nanopore sequencing, we used native barcodes on pooled amplicons and loaded 20–40 ng of library onto the flow cell. Samples from plates 5–8 were prepared for Nextera Flex Illumina sequencing as described:

https://www.protocols.io/view/sars-cov-2-mcgill-nextera-flex-sequencing-protocol-bisbkean. For samples sequenced on Illumina, the Nextera Flex kit was used starting from 150 ng of DNA following the procedure from the manufacturer. Plate 9 was sequenced using both Nanopore and Illumina technologies, as well as by applying the Cleanplex assay by Paragon Genomics, followed by MGI sequencing. For samples that were sequenced more than once, the data with the higher coverage was used to generate consensus sequences and subsequent phylogenetic analysis. For the Cleanplex assay, we used the Cleanplex for MGI SARS-CoV-2 research panel by Paragon Genomics. The assay utilizes 343 primer pairs tiled across the viral genome as described [20]. The manufacturer’s protocol (UG4002-1) was used, with the modification of increasing the multiplex PCR cycle number to 16 in order to improve the sequencing of samples with qPCR Ct values of > 29, followed by sequencing on an MGI DNBSEQ G400 instrument.

### Basecalling and consensus sequence generation

All samples were aligned to the reference genome of the Severe Acute Respiratory Syndrome Coronavirus-2 isolate Wuhan-Hu-1 (GenBank Accession MN908947.3) [21]. Aligned reads were then used to produce a consensus sequence using pipelines based on the Artic Network novel coronavirus bioinformatics protocol (https://artic.network/ncov-2019/ncov2019-bioinformatics-sop.html). A brief description of the pipeline, including software packages and important parameters, is provided for each sequencing platform below.

Datasets produced using the Nextera Flex Illumina protocol were first filtered to remove any host reads. To do so, reads were aligned to a hybrid reference including SARS-CoV-2 (MN908947.3) and GRCh38 using bwa-mem (v0.7.17) [22]. Any reads mapping to a region of the human reference with a mapping quality of zero or more were removed from the dataset. After filtering out host reads, the remaining reads were trimmed using cutadapt (v2.10) [23], then aligned to the SARS-CoV-2 reference (MN908947.3) using bwa-mem (v0.7.17) [22]. After alignment, reads were filtered using sambamba (v0.7.0) [24] to remove paired reads with an insert size outside of the 60–300-bp range, as well as any unmapped reads, secondary alignments and reads that did not match the FR/RF orientation. iVar (v1.3) [25] was used to trim any remaining primers. Samtools (v1.9) [26] was used to produce a pileup which was then used as input by iVar (v1.3) to create a consensus sequence for regions with a minimum of 10× depth, using reads with a Q score > 20 and a minimum allele frequency of 0.75. A full description of the process can be found here:

https://c3g.github.io/covseq_McGill/SARS_CoV2_Sequencing/Illumina_overview.html.

Datasets produced using the CleanPlex MGI protocol were processed using the same pipeline as Illumina Nextera Flex samples, except that Artic Network primers and amplicon data was changed to the corresponding CleanPlex information. A full description of the process can be found here:

https://c3g.github.io/covseq_McGill/SARS_CoV2_Sequencing/MGI_overview.html.

Raw data produced using Nanopore sequencing was basecalled using guppy (v3.4.4) [27] with a High-Accuracy Model (dna_r9.4.1_450bps_hac). Reads were de-multiplexed using guppy barcodes (v3.4.4), requiring barcodes on both ends. Reads were filtered by size to remove anything outside of the 400–700 bp range using the ARTIC Network “guppyplex” tool. Reads were aligned with minimap2 (v2.17) [28], then filtered to remove incorrect primer pairs and randomly downsample high-depth regions to keep 800× depth per strand using the ARTIC network framework. Nanopolish (v0.13.1) [29] was used to call variants in regions with a minimum depth of 16× and a flank of 10 bp. After masking regions with coverage below 20×, the called variants were used to generate a consensus sequence using bcftools (v1.9) [26] consensus. A full description of the process can be found here:

https://c3g.github.io/covseq_McGill/SARS_CoV2_Sequencing/ONT_overview.html.

For samples sequenced with two or more technologies, all datasets were processed separately using the methods described above. The resulting consensus sequences were compared to keep only the most complete consensus for downstream analyses, as determined based on the number of missing bases (Ns). After excluding consensus sequences with > 5% Ns, we were left with 2921 consensus sequences for further analysis (83.14% passing rate). The consensus sequences were deposited in GISAID under accession numbers listed in Additional file 2: Table S1. The raw sequence data is available in NCBI as described in the “Availability of data and materials” section below. The list of authors and laboratories for consensus sequences obtained from GISAID are in Additional file 3: Table S2.

### Positive and negative controls

Across our dataset, we included two types of negative controls and two types of positive controls:

- Extraction-negative controls (Ext controls) consist of a water blank included in every plate of samples before extraction. They are processed alongside the samples from RNA extraction onwards.
- Reverse transcription-negative control (RT control) consist of an extraction buffer blank included in plates before reverse transcription. They are processed alongside the samples from reverse transcription onwards.
- Viral culture-positive control consists of a sample of RNA extracted from a viral culture of B.1 lineage with 6 known SNVs. They are processed alongside the samples from reverse transcription onwards.
- Accukit RNA/cDNA positive control (Accugenomics Inc., Catalog number 1231) consists of artificial RNA or cDNA molecules with introduced SNVs every 90 bp. The artificial molecules do not cover the whole genome, and the RNA version covers 94% of the SARS-CoV2 genome whereas the cDNA version covers 83% of the SARS-CoV2 genome. They are processed alongside samples from reverse transcription (RNA) or right after (cDNA) depending on the kind of control used. For more information, consult the provider’s website at https://accugenomics.com/accukit-sars-cov-2/. Please be aware that for this dataset, a previous version of the kit was used; notably, the cDNA version of this kit is no longer available.

All cases detected before March 17, 2020, were processed in plates that included only extraction-negative controls and viral culture-positive controls. Samples detected after that date were processed in plates including all four controls (with only one of the two versions of the AccuKit control included). All controls were processed using the same bioinformatic pipelines as the rest of the samples.

For a plate to pass quality controls, extraction controls were not allowed to have more than 2× average depth of coverage or produce a consensus sequence covering more than 1% of the genome length. During processing, only two plates out of a total of 42 plates failed this test, and samples in those plates were repeated. Reverse transcription controls had the same threshold as extraction controls, but none of them exceeded these thresholds during processing.

Positive controls were evaluated based on coverage (consensus sequences of Accukit controls should not exceed their length based on the % of the genome covered by the kit) and by the detection of the known SNVs. All positive controls passed these evaluations.

### Phylogenetic analysis

Raw and time-scaled phylogenomic trees were built using the NextStrain pipeline (https://github.com/nextstrain, version 1.16.2) [30] installed in a conda environment (https://github.com/conda/conda, version 4.8.3). This pipeline uses the Augur toolkit (https://github.com/nextstrain/augur, version 7.0.2) [31] to filter, align/mask genomic sequences, build trees (divergence and time-scaled), and produce an output file processed by the Auspice web interface (https://github.com/nextstrain/auspice, version 2.16.0) [30] to explore phylodynamic and phylogenomic data. Augur removed all sequences shorter than 27,500 bp and sampled after June 1st, 2020. The Augur/align module was then called to execute the multiple sequence alignment with MAFFT (https://github.com/GSLBiotech/mafft, version v7.463) [32] using Wuhan-1 (Genbank accession MN908947) as a reference genome. The final alignment was masked at the beginning (first 100 sites) and end (last 50 sites), and at positions 18529, 29849, 29851, and 29853 (sites of known low sequencing quality and homoplasies). We then used Augur to select sequences from GISAID that were most similar to our 2921 Québec sequences. These global context sequences were then grouped by country/month in order to keep a maximum of 100 sequences and 5 identical sequences per country-month combination. In this way, a total of 12,801 genomes were pulled from GISAID on October 30th, 2020 (Additional file 3: Table S2).

We used IQ-TREE (http://www.iqtree.org/, version 1.6.12) [33] to construct a phylogenetic tree of Québec only sequences and another tree of Québec and global context sequences, with the GTR substitution model. Branch lengths, sampling dates, and ancestral states (geographic regions, nucleotides and amino acids sequences) at internal nodes were inferred with the Augur/refine and Augur/traits modules by calling TreeTime (https://github.com/neherlab/treetime, version 0.7.5) [34] (using the same default parameters as those chosen in public builds; https://github.com/nextstrain/ncov). Finally, the Augur/export module exports a single compiled results file required for data visualization in Auspice. All Nexstrain analyses were executed on a 64-bit CentOS server version 7.4.1708 using 40 CPUs.

Clade assignment was done during the NexStrain build. As input, the Augur/clades module uses the phylogenetic tree, the observed and inferred nucleotide sequences at each node and a clade configuration file. In this clade file, every single clade value is associated with a specific combination of position/nucleotide variant. As an alternative clade assignment scheme, we also used the Phylogenetic Assignment of Named Global Outbreak LINeages [12] combined with lineages (https://github.com/cov-lineages/lineages) version 2020-05-09 (Pangolin 2.3.2 and pangoLearn 2021-02-21, https://cov-lineages.org/pangolin_docs/pangolearn.html).

To infer introduction events into Québec (QC), we used discrete character ancestral state reconstruction (ASR) to infer non-QC and QC nodes in the global context time tree. Three methods were implemented in *R* [35] using either (1) maximum likelihood (*ace* function from *ape* package v5.4-1 [36], assuming the equal rates model), or unordered Fitch parsimony implemented with the *fitch.mvsl* function in *mvSLOUCH* v2.6.1 [37] either with (2) delayed (DELTRAN) or (3) accelerated (ACCTRAN) transformation algorithms in order to deal with ambiguous nodes. With the reconstruction, we assigned nodes to the QC state when supported by ≥ 50% (with ML) or 1 (with parsimony) of the state assignment. To find the transitions from non-QC to QC nodes, first we identify every QC node or tip that is preceded by a non-QC node. Next, among this set of nodes, we look for the most basal and discard every node that is a descendant of another node in this set. The parents of these remaining nodes are the non-QC node of the transition and the introduction event is considered to have happened within the transition. Note that these methods likely underestimate the number of introductions. The non-QC to QC transitions were collected and their most basal QC leaf (or leaves) are recorded. These candidates were then cross-checked with travel history data and were only recorded if at least one had travel history. In the case of genetically identical genomes in two travellers with the same travel history, we assumed only one introduction event, which is a conservative estimate, given that in principle both could cause secondary infections in Québec. If only using travel history, they would be counted as two separate introductions. In the case of a polytomy with multiple basal QC sequences, only one was chosen by the shortest branch length. If no travel history was available, then the closest outgroup of the introduction event was used to assign the likely origin of the introduction event. The descendants of these identified transitions were used to define QC transmission lineages. The date of the non-QC node (prior to the first QC node) was used as the TMRCA of the introduction event (Additional file 1: Figure S1). For the largest QC transmission lineages (containing > 20 cases), the TMRCA was also inferred using BEAST (see below). Phylogenetic visualizations and dataset manipulation were done in R using a suite of packages: *ape* [36], *phylotools* [38], *phytools* [39], *phangorn* [40], *tidyverse* [41], *ggtree* [42], and *treeio* [43].

### Phylodynamics

The molecular clock signal was assessed by plotting the root-to-tip phylogenetic distance against time using TempEst [44]. The largest (> 20 sequenced cases) QC transmission lineages were analyzed using Bayesian phylogenetic tree reconstruction with Markov chain Monte Carlo (MCMC) implemented in BEAST v2.6.2 [45] with the Birth-Death Skyline (BDSKY) model, assuming a gamma distributed Hasegawa-Kishino-Yano (HYK) nucleotide substitution model (with a uniform distribution 0.25 [0,1] of the nucleotide frequencies, a lognormal 2 [0, ∞] for □, and a □ count of 4 with an exponential distribution 0.5 [0, ∞]), and a strict molecular clock (0.8 × 10^−3^ substitutions/site/year). Using this model, we estimated the effective reproduction number (*R*_*e*_), TMRCA, and sampling proportion. The prior for the reproduction number was a lognormal distribution (initial = 2 [0,10], *M* = 0, *S* = 0.5), origin was a normal distribution (mean = 0.1, □ = 0.05, initial 10[0,∞]), the rate of becoming uninfectious was a normal distribution (mean = 10, □ = 1.3, initial = 1[0,∞]), and sampling rate was a beta distribution (□ = 1, □ = 5, initial = 0.01[0,1]). All MCMC analyses were run with 50 million generations and sampling every 50,000 steps for lineages with > 100 cases and 30 million generations and 30,000 steps for lineages < 100 cases. Convergence was achieved when all posteriors gave effective sample sizes (ESS) > 300 with 10% burn-in.

### Calculation of population genetic metrics

We calculated Tajima’s *D* to infer changes of the viral effective population size and deviation from a standard neutral evolutionary model. We separated the data into eight time periods of 2 weeks between February 20, 2020, and June 10, 2020. For each time period, we randomly sampled 20 viral consensus sequences to calculate Tajima’s *D*, and repeated this procedure 99 times to obtain confidence intervals. We calculated both a combined value of *D* across all sequences, and a separate estimate for each PANGO viral lineage. Lineages or time bins with fewer than 20 sequences were discarded. We calculated *D* as described [46]:

1 $$ {D}_{\mathrm{Tajima}}=\frac{\theta_{\pi }-{\theta}_w}{\sqrt{\hat{V}\left({\theta}_{\pi }-{\theta}_w\right)}} $$

where the $\hat{V}$ denotes the expected sampling variance of (*θ*_*π*_ − *θ*_*w*_). *θ*_*π*_ is the nucleotide diversity, calculated based on the average number of pairwise differences among consensus sequences:

2 $$ {\theta}_{\pi }=\frac{Nb\_ reads\_ pwdiff}{\sum_{i=1}^nC\left(N,2\right)} $$

where *n* is the genome length, *N* is the number of consensus sequences, *C*(*N*, 2) is the choose() function which calculates the number of pairs of consensus sequences in a set of size *N*, and *Nb_reads_pwdiff* is the number of pairwise nucleotide differences. Because pairwise differences are maximized when there are intermediate-frequency mutations, *θ*_*π*_ is more sensitive to intermediate-frequency mutations. *θ*_*w*_ is another estimator of the nucleotide diversity which is calculated based on the number segregating sites and is sensitive to low-frequency mutations:

3 $$ {\theta}_w=\frac{S}{a_1} $$

4 $$ {a}_1={\sum}_{i=1}^{N-1}\frac{1}{i} $$

where *S* in the number of segregating sites, *a*_1_is a normalizing factor for the sample size of consensus sequences (*N*).

We also estimated *dN/dS*, the ratio of nonsynonymous (*dN*) and synonymous substitutions rates (*dS*), by comparing consensus sequences to the reference genome (Genbank MN908947.3) allowing us to infer changes in selective pressures at the protein level.

5 $$ \frac{dN}{dS}=\frac{\left(N{b}_{nsub}/N{b}_{nss}\right)}{\left(N{b}_{ss ub}/N{b}_{ss}\right)} $$

where *Nb*_*nsub*_ is the number of nonsynonymous substitutions, *Nb*_*nss*_ is the number of nonsynonymous sites, *Nb*_*ssub*_ is the number of synonymous substitutions, and *Nb*_*ss*_ is the number of synonymous sites. We only considered consensus sequences with more than 1 synonymous mutation to be able to attribute finite values to *dN/dS*. These analyses were coded in R [35].

## Results and discussion

### Sampling and sequencing the first wave in Québec

Our province-wide sequencing effort covers the first pandemic wave up to June 1st, 2020, with a focus on the earliest confirmed cases up to April 1st (Fig. 1A). No particular outbreaks were targeted, in an effort for an approximately random sequencing of qPCR-positive swab samples (without any cycle threshold cutoff; “Methods”). Consensus sequences of SARS-CoV-2 viral genomes were obtained by targeted amplification from clinical nasopharyngeal swabs specimens followed by sequencing on Nanopore (*n* = 180), Illumina (*n* = 2630), or MGI (*n* = 111) platforms. Only sequences passing quality criteria (less than 5% undetermined bases, “Ns”) were considered for further phylogenetic analyses (“Methods”; Additional file 2: Table S1). With these genome sequences, we covered 5.7% of the total number of reported cases (45,641 laboratory confirmed cases and 5849 suspected cases) up to and including June 1st. To capture early introduction events, our sequencing effort was highest (covering 27% of cases) before April 1st, 2 weeks after the Canadian border closed and most repatriation of Canadian citizens from abroad had occurred. Until early April, the mean age of sequenced cases was approximately 50 years old, then jumped to ~ 75 years old, likely reflecting that the virus had entered long-term care facilities (Fig. 1B). By April 1st, over 500 long-term care facilities had reported at least one case of COVID-19, and the virus spread steadily through these primarily elderly populations during the month of April [47].

### Inferred SARS-CoV-2 introductions to Québec are mostly of European origin

Before federally mandated quarantine orders for returning travellers were put in place on March 25th, 1544 travellers who entered Québec had tested positive for SARS-CoV-2 [17]. However, not all these cases were necessarily independent introduction events, nor would they all give rise to successful onward transmission of SARS-CoV-2. To complement and refine the identification of introduction events, we compared self-reported travel history provided by qPCR-positive COVID-19 cases with a phylogenetic inference of Québec and global context sequences (*n* = 15,722 viral genomes from GISAID; Additional file 3: Table S2). Following previous phylogenetic studies of SARS-CoV-2 [2, 5], we used ancestral state reconstruction (ASR) to identify non-Québec to Québec transition nodes in the phylogeny (Additional file 1: Figure S1 and “Methods”). In this way, we inferred a total of 615 independent introduction events based on maximum likelihood (ML) ASR and 579 to 682 introductions based on ACCTRAN and DELTRAN parsimony ASR, respectively (Additional file 1: Figure S2 and Additional file 4: Table S3). We will refer to ML ASR results below unless otherwise indicated, and ranges refer to results of all three methods. In our preliminary study of 734 Québec sequences up to April 1st, we estimated only 247 introduction events [48], suggesting that introductions are underestimated and are likely to increase with sample size. Here we report a sample of genomes 4 times larger than our preliminary study, but the number of introductions is only 2.5 times higher, suggesting a plateau in inferred introductions. We defined Québec transmission lineages as descendants of a unique introduction event in the phylogeny, and then annotated these based upon Pangolin (PANGO) [12] and Nextstrain [30] lineage nomenclatures. Note that PANGO or Nextstrain are viral phylogenetic lineages used for taxonomic purposes, which are distinct from Québec transmission lineages, which we define at higher phylogenetic resolution as descendants of a single introduction event, and thus represent a partially observed transmission chain.

We calculated Tajima’s *D* as a simple non-parametric metric of viral effective population size [49] and found strongly negative values of *D* early in the epidemic, consistent with exponential growth in mid-March to early April, followed by decelerating growth as public health measures likely reduced viral transmission (Fig. 1C). The decline of Tajima’s *D* from March 5 to 19 coincides with, or slightly precedes the increase in the epidemic curve starting on March 19th, suggesting its utility as an early indicator of population expansion. For example, PANGO lineage B.1, which originated in Italy and spread throughout Europe, showed evidence of rapid growth in Québec (median *D* ~ − 2.5 in mid-March) followed by a decline in late April and May. This is consistent with our observation that B.1 became very common in Québec by April before being replaced by other B.1 variants (notably B.1.147 and B.1.350) by the end of May (Additional file 1: Figure S3).

**Fig. 2:**
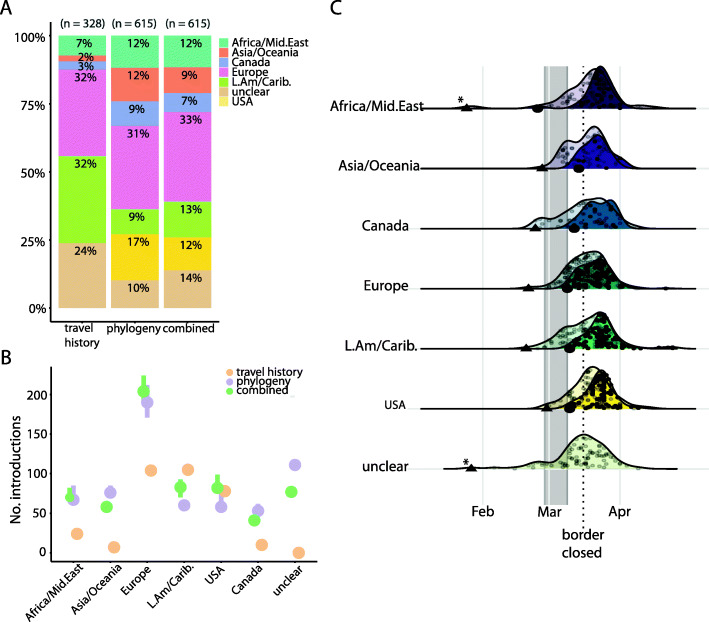
Analysis of introduction events. **A** Introduction event origins as a percentage of the total inferred by each method. **B** Number of introduction events by region of origin inferred by self-reported travel history, phylogenetic ancestral state reconstruction, or both combined. For phylogeny-only and combined estimates, the points represent the ML estimate, while the bars indicate the difference between DELTRAN and ACCTRAN estimates (as lower and upper bounds). “Canada” refers to importations from other provinces into Québec. “Unclear” implies no travel history was available and ASR was ambiguous. **C** Travel-related sequences and the TMRCAs of inferred introductions into Québec over time by geographic origin. *Dark densities*: small points indicate sampling dates of sequenced cases with travel history. Large black points indicate the sampling date of the first sequenced case associated with each region. *Pale densities*: small points indicate the TMRCA of the inferred introductions using phylogeny and travel history (thus the date of introduction into Québec will be later). Triangles are the TMRCA of the first inferred introduction from each region into Québec, based on the phylogeny. Asterisks indicate uncertainty due to stem singletons in a large polytomy. The number of introductions is normalized to a relative density within each geographic category (rows). Grey bar is the spring break period in Québec

Of the 2921 Québec consensus sequences analyzed here, 328 were from COVID-19 cases that had reported recent travel history. Note that a lack of travel history could indicate a true lack of travel, or a lack of available data. Travellers reported returning from the Caribbean and Latin America (*n* = 105, 32%, mainly from Mexico, *n* = 31, 9.5% and the Dominican Republic, *n* = 30, 9%), Europe (*n* = 104, 32% with the most from France, *n* = 39, 12% and Spain, *n* = 20, 6%), and the USA (*n* = 77, 24%) (Fig. 2A). There was very little reported travel from Asia (*n* = 4, 1.2%) and none from China. A moderate percentage of the phylogenetically inferred introduction events (25–28%; range across parsimony or ML methods) were associated with travel history. This is consistent with travellers being encouraged to get tested, along with the high sequencing coverage in the early days of the pandemic in Québec (Fig. 1A). These events were broadly concordant with travel history, with some exceptions: notably, Latin America and Europe were approximately equally popular destinations based on travel history, but phylogenetic analysis identified Europe as the more likely origin of introductions into Québec (Fig. 2A, B). This is consistent with European PANGO lineages arriving in Québec, perhaps via the Americas—but before accumulating lineage-defining mutations in the Americas. The early introductions of PANGO lineages A and B.4, common in the early outbreaks in China and Iran respectively, appear not to have been successful in Québec and were not observed by mid-May (Additional file 1: Figure S3).

### Successful cryptic introductions before the first reported case are unlikely

The first confirmed case of COVID-19 in Québec was detected on February 25th, but phylogenetic analysis has the potential to infer earlier introduction events. We inferred 15–17 potential introduction events before February 25th, based on their time to the most recent common ancestor (TMRCA), of which only 5–6 had reported travel history. Throughout, we refer to the TMRCA of the divergence between a Québec lineage and its closest non-Québec outgroup (Additional file 1: Figure S1). The introduction event into Québec must have happened after the non-QC TMRCA and before the first sequenced case in QC (Additional file 1: Figure S1) and thus the earliest possible introduction time would be the TMRCA. As expected, the TMRCA estimates thus tend to be earlier in time compared to the sampling dates of returning travellers (Fig. 2C). The TMRCA estimates are similar among the three ASR methods applied to the TreeTime phylogeny, but are consistently later when compared to estimates from BEAST (Additional file 1: Figure S2). Because BEAST more thoroughly accounts for phylogenetic uncertainty than TreeTime, we use the BEAST TMRCA estimates for detailed analyses of large transmission lineages below.

The global phylogeny during the first 2 months of 2020 is undersampled, due to sequencing efforts only beginning to ramp up at that time. This, combined with relatively slow accumulation of mutations by SARS-CoV-2, resulted in many large polytomies (unresolved branchings), making precise inference of introductions challenging. Indeed, ten of these 15–17 early introductions are in polytomies and 33–40% are of unclear origin; thus, their true TMRCA is questionable (Fig. 2C). For example, the first TMRCAs from Africa/Middle-East and those of unclear origin are in large polytomies and thus their true introduction events are likely to be later than inferred in the time-scaled ML tree (cases marked with asterisks in Fig. 2C). The earliest reliably dated introduction has a TMRCA of February 15th, from Europe (clustering with sequences from Switzerland), followed by an introduction from the UK with a TRMCA of February 21st. We do not reliably detect any introductions arriving in January or early February, which is consistent with a study of samples from patients with flu-like symptoms between November 2019 to early March that did not find any SARS-CoV-2, suggesting that introductions before late February are unlikely (as reported in *Le Devoir*, September 5th, 2020 [50];) and appear not to have given rise to sustained transmission.

### Most introductions occurred after spring break

To test the hypothesis that spring break travel was a major source of viral introductions into Québec, we investigated the Québec transmission lineages with a TMRCA between Feb. 23rd and March 10th and defined them as having been plausibly introduced during spring break. Given that TMRCAs provide an early bound for transmission into Québec (Additional file 1: Figure S1), we included introductions with a TMRCA of Feb. 23rd to account for a lag of up to 6 days from infection to sampling. During this period, there were 80–100 introduction events (only 12–16% of the total), of which 29–37 had recorded travel history (~ 37%). The majority of introductions likely happened after spring break, with 77–83% of TMRCA estimates after March 10th (Fig. 2C and Additional file 1: Figure S2). The USA is a common travel destination for Québecers, where many (known as “snowbirds”) have winter homes. The bulk of the USA travel-related cases were detected after the border closed on March 16th, and thus were likely part of the repatriation effort. However, the phylogenetically inferred introductions from the USA suggest that these were not as successful as the introductions that happened in early March: the only transmission lineages with > 20 viral genomes of US origin arrived before March 15th (Fig. 2C). The majority of the 41–48 introductions from other Canadian provinces were not reported in travel history records (38–45 introductions, 93–95%), which is consistent with inter-provincial travel having been common until being discouraged in late March.

### Successful transmission lineages arrived early and spread widely

**Fig. 3:**
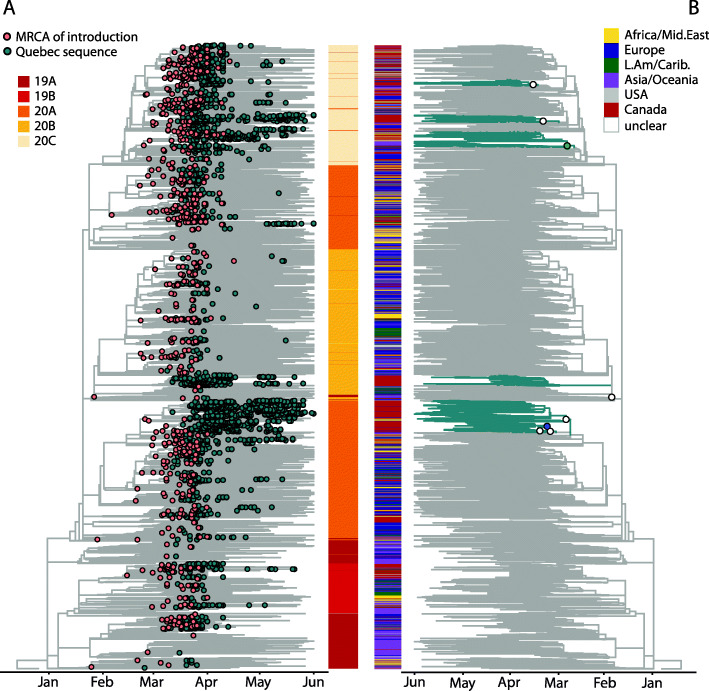
Phylogeny of SARS-CoV-2 genomes sampled from Québec in global context. **A** Pink dots on the time-scaled phylogeny show the most recent common ancestors (MRCA) of introduction events into Québec, inferred with ML ASR. Blue dots are all Québec sequences in the dataset. The heatmap shows Nextstrain clade designations for all sequences in the tree. **B** The same phylogeny, highlighting (blue branches) the eight Québec transmission lineages that gave rise to over 50 sequenced cases. Their introductions (large circles) are colored by their inferred region of origin. The colored heatmap shows the geographic origin of all sequences in the tree

**Fig. 4:**
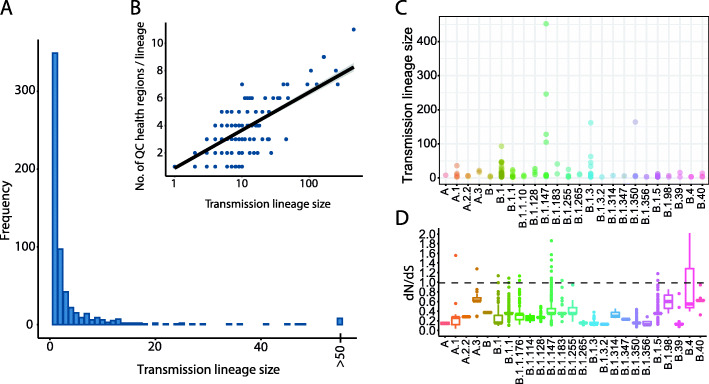
A minority of introduction events are successful and spread across regions. **A** Distribution of Québec transmission lineage sizes, inferred using maximum likelihood ASR, *n* = 615. **B** Correlation between transmission lineage size and the number of Québec health regions in which it was sampled (Pearson’s *r* = 0.62, 95% CI = 0.57–0.67, *p* < 2.2 × 10^−16^). **C** Transmission lineage sizes generated by each PANGO lineage. Each point represents an independent introduction event into Québec. **D** Estimates of *dN/dS* for each PANGO lineage in Québec. For each PANGO lineage, the boxplots represent the distribution of *dN/dS* across sampled genomes compared to the ancestral reference genome (Wuhan-1; Genbank Accession MN908947.3)

Of the total 579–682 independent inferred introduction events, the majority were singletons (52–63% with only one observed sequence). Our ~ 5% sequencing coverage implies that many of these singletons might actually represent small transmission lineages of around ~ 20 cases (assuming undersampling is uniform across lineages of all sizes). Similarly, the 72–76% inferred introductions that gave rise to small transmission lineages of less than 3 sampled genomes, could each represent transmission lineages of up to 40 cases. In contrast, only 7–12 introductions (0.9–1.6% of the total; range of estimates from parsimony and ML) were successful enough to cause more than 50 sequenced cases in Québec (Fig. 3), which implies likely transmission lineages of approximately 1000 cases. The top eight introductions inferred by ML gave rise to 1544 genomes, or 53% of all sequenced cases (ASR parsimony range: 7–12 introductions giving rise to 52–75% of all sequenced genomes in the first wave) until June 1st. This overdispersion is more extreme but qualitatively similar to a UK study where the eight largest introductions resulted in > 25% of cases [2]. This highly overdispersed transmission lineage size distribution (Fig. 4A) is also similar to what was observed in Massachusetts [5]. These results are consistent with an overdispersed reproductive number and suggestive of superspreading, in which most potential transmission events are unsuccessful but a minority give rise to dozens or hundreds of subsequent infections.

Larger transmission lineages tended to be sampled across more regions in Québec (Fig. 4B), indicating that the success of these transmission lineages was not due only to local outbreaks but rather to wide geographic spread across the province. The PANGO lineages that spread the most throughout Québec (i.e., being found in more than ten health regions) were B.1, B.1.5, and B.1.147 (Additional file 1: Figure S4). Similar to another study of SARS-CoV-2 in Canada [51], we find that B.1 was by far the most introduced PANGO lineage in Québec (introduced 216 times, 51% of which were singletons). PANGO lineage B.1.147 stayed mostly in the more populous southern regions of the province while genomes of B.1 were found in almost all regions (Additional file 1: Figure S4 and Additional file 1: Figure S5). Lineage B.1.5 first arrived after spring break and was introduced 41 times across Québec but was not successful at generating transmission lineages of over 12 sampled genomes and was no longer observed by June 1st (Additional file 1: Figure S3 and Additional file 1: Figure S4). In contrast, B.1.147 was introduced half as many times (19 introductions) but these events tended to have occurred earlier in spring break (Fig. 4C and Additional file 1: Figure S3).

The most successful Québec transmission lineages belonged to B.1.147, B.1.350, B.1.3, and B.1, each of which was introduced multiple times (Fig. 4C, Additional file 1: Figure S4, Figure S5, and Figure S6). The prominent spread of B.1.147 and B.1.350 in Québec is unique compared to the rest of Canada (Additional file 1: Figure S3). In contrast, PANGO A lineages were more common in British Columbia (consistent with more initial transmission from Asia) and B.1.1 sublineages in Ontario [51]. The PANGO lineages common in Québec (B.1.147, B.1.350, B.1.3, and B.1) had evolutionary rates comparable to other lineages in Québec (~ 6 × 10^−4^ substitutions per site per year; Additional file 1: Figure S7), somewhat slower but in the range estimated from other studies [52]. To quantify variation in the strength of natural selection on these PANGO lineages, we calculated the nonsynonymous to synonymous substitution ratio (*dN/dS*) between all pairs of genomes within a PANGO lineage (Fig. 4D). Note that this simple estimate of *dN/dS* does not account for multiple substitutions at the same nucleotide site. Over the short evolutionary time scale considered, we do not expect this to significantly affect the results. There was a modest positive correlation between a PANGO lineage’s *dN/dS* and its average transmission lineage size (Pearson’s *R*^*2*^ = 0.12, permutation test *P* = 0.0002). Much of this correlation is driven by lineage B.1.147 (Fig. 4C, D) and could be explained by the accumulation of low-frequency, slightly deleterious nonsynonymous mutations at the tips of a rapidly expanding clade [53, 54], which is also consistent with strongly negative Tajima’s *D* values (Fig. 1C). Together, these results suggest rapid population growth of the most successful PANGO lineages.

**Fig. 5:**
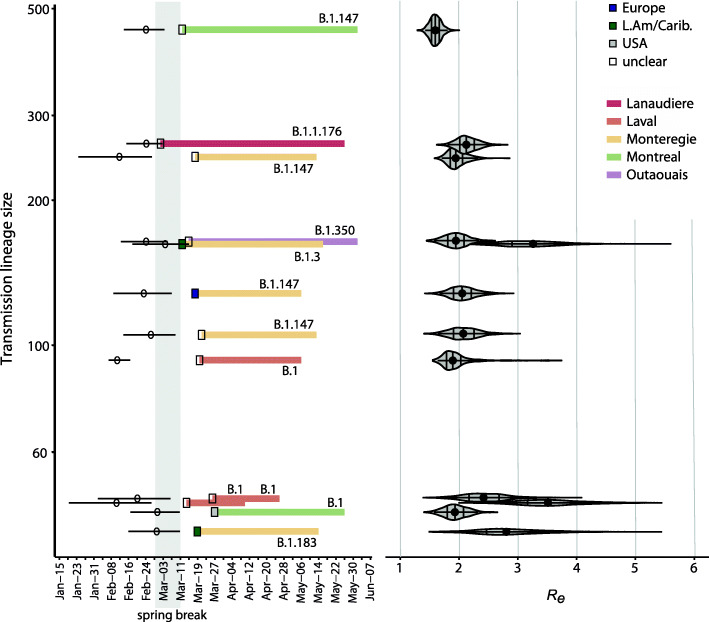
Introduction and duration of the 12 largest Québec transmission lineages each responsible for 40 or more sequenced cases. Left: Timelines are colored by the health region in which each Québec transmission lineage predominantly spread, and which SARS-CoV-2 lineage (Pangolin nomenclature) was responsible. Squares represent the date the lineage was first sampled, colored by their inferred origin. Open circles show the median TMRCA of each transmission lineage, with whiskers showing HPD 95% intervals estimated with BEAST. Right: median and HPD 95% interval of the effective reproductive number, *R*_*e*_, for each transmission lineage, estimated using BEAST (Methods). These transmission lineages were inferred by ML ASR. See Figure S2 for TMRCA estimates for the 21 largest transmission lineages, represented by at least 20 sequences

The 7–12 largest transmission lineages likely arrived relatively early (all TMRCA 95% HPD before or during spring break) and were still detectable in late May (Fig. 5). The median effective reproductive numbers (*R*_*e*_, estimated by phylodynamic analysis) for 10 of the 12 largest transmission lineages were estimated in the range of 2–3, consistent with exponential growth (Fig. 5). Two transmission lineages by B.1 and B.1.3 had higher *R*_*e*_ values, potentially due to their rapid spread in long-term care facilities. Although our dataset lacks explicitly defined outbreaks in care facilities or elsewhere, we used the following criteria to identify likely care facility outbreaks caused by large transmission lineages (with at least 20 sequenced cases): a monophyletic group of three or more seniors (at least 60 years old), similar date of sampling (within 5 days), and identical Québec health region. We counted an average of 1.5 possible care facility outbreaks per large transmission lineage (SD 1.6, range 0–6). This suggests that, even if our sampling did not directly target these outbreaks, they are well-represented in the dataset. The B.1 transmission lineage that spread mostly in a care facility in the city of Laval [55], is a particularly striking example, where the median age jumped to 83 years old (IQR 71 to 89 years) after a likely introduction by a person in their forties (Additional file 1: Figure S8). This outbreak was brought under control, and no sequences were detected past early May (Fig. 5; Additional file 1: Figure S8).

The self-isolation mandates for arriving travellers (Québec’s orders on March 11th and federal mandatory quarantine orders on March 25th) appear to have been effective, such that there were more larger transmission lineage sizes that started before March 11th than after March 11th (skewness of the lineage size frequency distribution pre-March 11th = 6.25 vs skewness post-March 11th = 9.42; pre-March 11th range = [1, 452], post-March 11th range [1, 105]). After the federal quarantine orders, 70% of introductions were singletons and only four gave rise to 10 or more sampled genomes. The TMRCA of the last introduction event inferred in our dataset was April 16th, 2020.

### Mutation and founder effects on transmission lineage size

**Fig. 6:**
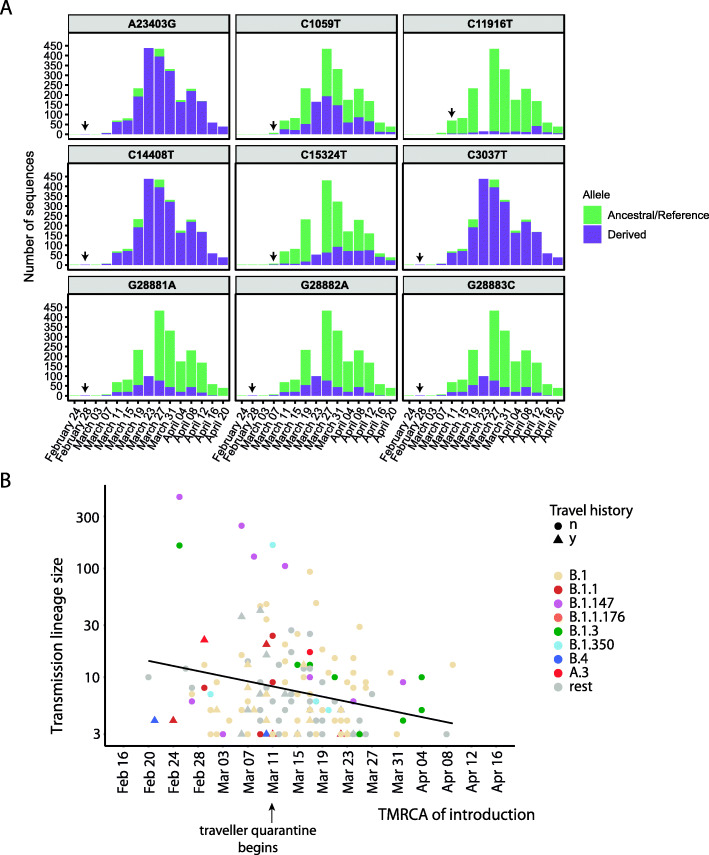
Arrival of SARS-CoV-2 lineages and transmission-associated mutations over time. **A** Number of consensus sequences including each lineage-defining mutation, named by alternative nucleotides and genome position. The first detected occurrence of each derived allele is indicated by an arrow. The nucleotide positions correspond to the following amino positions in genes: A23403G;D614G;S;S, C1059T;T85I;orf1a;NSP2, C11916T;S25L;orf1a;NSP7, C14408T;P323L;orf1b;NSP12, C15324T;N629N;orf1b;NSP12, C3037T;F107F;orf1a;NSP3, G28881A;R203K;N;N, G28882A;R203R;N;N, G28883C;G204R;N;N. Nucleotide positions are coded as follows: ancestral nucleotide allele, genome position, derived nucleotide allele; ancestral amino acid allele, position in protein, derived amino acid allele; ORF; gene. **B** Introductions that generated transmission lineages of > 2 genomes as a function of the TMRCA (inferred by ML ASR), colored by PANGO lineage name and annotated if the introduction had reported travel history (y) or not (n). Pearson correlation coefficients: *r* = − 0.30 (− 0.43, − 0.16), *p = 6.828e−05*, black line. Note that the last inferred introduction event had a TMRCA of April 16th, not shown here because it was a singleton

Finally, we considered the extent to which the success of an introduction event could be explained by founder effects or adaptive mutations. To investigate the role of specific mutations, we defined nine lineage-specific single nucleotide variants (alleles) present in all members of each PANGO lineage and tested their associations with transmission lineage size. We found that mutation D614G in the Spike protein (genome position A23403G) was present in all ten of the most successful introduction events into Québec (Additional file 1: Figure S9) and generally dominated our sampled sequences (Fig. 6A). Independent introductions of PANGO lineages with the derived G allele gave rise to a mean transmission lineage size of 6.6 cases, compared to 3.4 for the ancestral D allele; however, this difference is not statistically significant (Additional file 1: Figure S9). In contrast, derived nonsynonymous mutations in three consecutive nucleotide sites (28881-28883) spanning two codons in the nucleocapsid (N) protein were significantly associated with smaller transmission lineage size (Additional file 1: Figure S9) and were less represented in our sequences (Fig. 6A).

If founder effects also played a role in determining successful transmission, we would expect the earliest introduction events to give rise to larger transmission lineages. Consistent with founder effects, we observed a significant negative correlation between inferred arrival time and transmission lineage size (Fig. 6B). This negative correlation was also observed using alternative ASR methods (Additional file 1: Figure S10). We also note that most of the early, successful introduction events had no reported travel history, highlighting the importance of phylogenetic analysis in identifying them. Therefore, while we cannot exclude a role of specific mutations affecting transmission, lineage success in Québec’s first pandemic wave can most parsimoniously be explained by a combination of founder effects and effective public health measures.

## Conclusions

Québec is unique among Canadian provinces for its early spring break, which resulted in many returning travellers before border closures and quarantine measures were enacted. Mathematical modelling results [17] suggest that Québec’s large SARS-CoV-2 first wave was, in part, driven by an early spring break. Here we show that earlier introduction events were much more likely to give rise to sustained transmission, compared to less successful later arrivals. Even though most introduction events were inferred after spring break, those that arrived before or during spring break generated the largest transmission lineages. Before quarantine and other public health measures were in place, eight introductions that arrived during spring break gave rise to hundreds of subsequent infections and spread widely across Québec. While hundreds of introduction events continued to occur after spring break, these spread much less widely, likely due to effective public health measures. This scenario closely mirrors the early SARS-CoV-2 introductions into the UK, which also spread widely and proved hard to eliminate [2].

Our phylogenetic analysis is generally concordant with self-reported travel history, but also revealed a large number of introduced European SARS-CoV-2 PANGO lineages that were not apparent from travel history. Québec is, thus, similar to other East Coast North American epidemics, such as Boston [5] and New York [56], that were primarily seeded by European lineages, in contrast to more Asian lineages on the West Coast [8, 51]. The Québec sequences were distributed broadly across the global phylogenetic tree, representing most of the known diversity, with an under-representation of early-branching Asian lineages. Like other phylogenetic studies, ours is limited by sampling: we cannot reliably detect introduction events from countries poorly represented in public databases, nor have we sequenced all SARS-CoV-2 infections in Québec. We were able to sequence ~ 6% of positive cases, putting our effort nearly on par with other leading genomic surveillance projects (e.g., ~ 8% in the UK as of spring 2021; https://www.cogconsortium.uk/). Nevertheless, our estimate of approximately 600 independent introduction events is almost certainly an underestimate. This highlights the need for sustained genomic surveillance efforts.

Although it is notoriously difficult to disentangle demographic factors from fitness effects of viral mutations [57], our results are consistent with a mild (not statistically significant) transmission advantage of the D614G Spike mutation, as observed elsewhere [10]. We also identified three adjacent derived mutations in the N protein associated with smaller transmission lineage size. These mutations (nucleotide positions 28881-28883) have been reported before, but their functional significance remains unclear [58] and could warrant further study. While these mutations may have played some role in affecting transmission in Québec, the differential success of introduced lineages is parsimoniously explained by founder effects, such that the first PANGO lineages that arrived tended to be successful. The recent success of lineage B.1.1.7 (alpha variant), which spread in the UK and elsewhere despite competition from previously established PANGO lineages, cannot be easily explained by founder effects [13]. Nevertheless, founder effects and other demographic factors must be carefully considered when inferring a transmission advantage of PANGO lineage of interest.

We observed an overdispersed distribution of introduced transmission lineage size: most introduction events went extinct, while only 7–12 introductions (< 2%) gave rise to at least one third of sequenced cases. Although we did not directly document specific superspreading events, the overdispersed distribution of lineage sizes is consistent with superspreading dynamics, as documented previously using genomic epidemiology [5]. Viral lineages that were introduced after the self-isolation mandate for travellers were largely unsuccessful at generating large transmission lineages, despite repeated introductions into Québec. Although our province-wide sampling was not designed to focus on specific outbreaks, they are reflected in our dataset. For example, one introduction of viral lineage B.1 during spring break quickly spread from younger to older individuals in a long-term care facility. This example mirrors the broader trajectory of the Québec epidemic in the first pandemic wave. Our study demonstrates the importance of timely public health actions during the early phases of a pandemic and how they shape the dynamics, size, and geographical spread of a novel pathogen.

## Supplementary Information

**Additional file 1: Document with Figures S1 – S10.**

**Additional file 2: Table S1.** List of Quebec sequence identifiers.

**Additional file 3: Table S2.** List of GISAID sequences and author acknowledgements.

**Additional file 4: Table S3.** Inferred introduction events using three ASR methods without travel history.

## Data Availability

Sequences we generated are available in GISAID and VirusSeq Portal https://virusseq-dataportal.ca/explorer (Additional file 2: Table S1) and the raw sequence data is available in NCBI under BioProject PRJNA686074, https://www.ncbi.nlm.nih.gov/bioproject/686074 [59]. This dataset is also available on our consortium’s website here: https://covseq.ca/data-info?lang=en. For all international genomes from GISAID used in this project, see Additional file 3: Table S2 for IDs and lab acknowledgements. All code for producing the table of inferred introductions and figures is available at https://github.com/murallcl/CoVSeQ_introductions [60]. Non-sensitive metadata is also provided. All SOPs generated and used by the CovSeQ consortium are found here: https://c3g.github.io/covseq_McGill/SARS_CoV2_Sequencing/about.html and workflows are also described on our website here: https://covseq.ca/methods?lang=en
