# Supplementary material for: A small number of early introductions seeded widespread transmission of SARS-CoV-2 in Québec, Canada
Source: Genome Med. 2021 Oct 28;13:169. doi: 10.1186/s13073-021-00986-9 (PMC8550813; doi:10.1186/s13073-021-00986-9)
Supplement: Supplementary file 1 — Additional file 1: Document with Figures S1 – S10. [file 13073_2021_986_MOESM1_ESM.pdf]

Supplementary Material

Additional file 1

Murall et al. 2021 “A small number of early introductions seeded widespread transmission of SARS-CoV-2 in Québec, Canada” Genome Medicine

Supplementary Figures

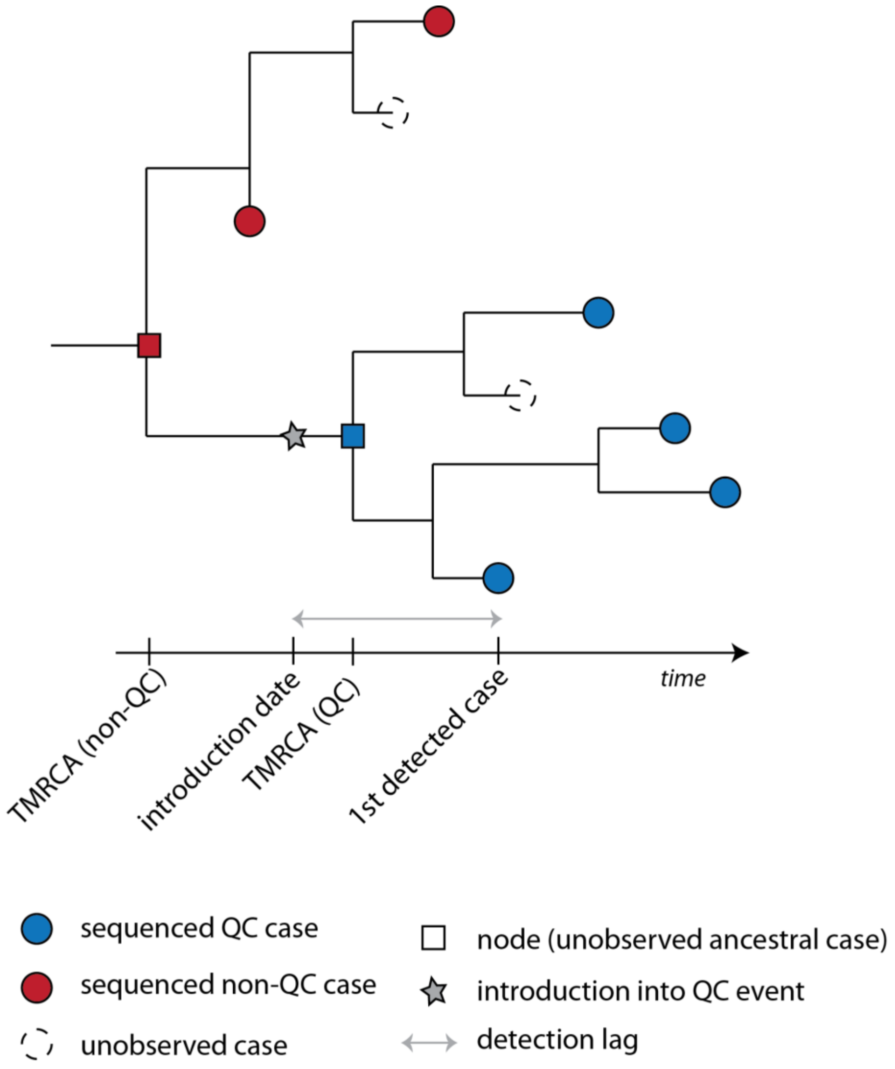

Figure S1. Schematic of an inferred QC transmission lineage. Our ASR method identifies transitions from non-QC to QC nodes on the time trees containing both QC and international sequences. The introduction event is believed to have happened in the time between the non-QC and QC nodes on the tree. Note that the detection lag is between the introduction event and the date of sampling of the first QC case. In order to assign origin, we would use the travel history of the first detected case in QC, but if they did not have travel history then we used the location assigned to the outgroup.

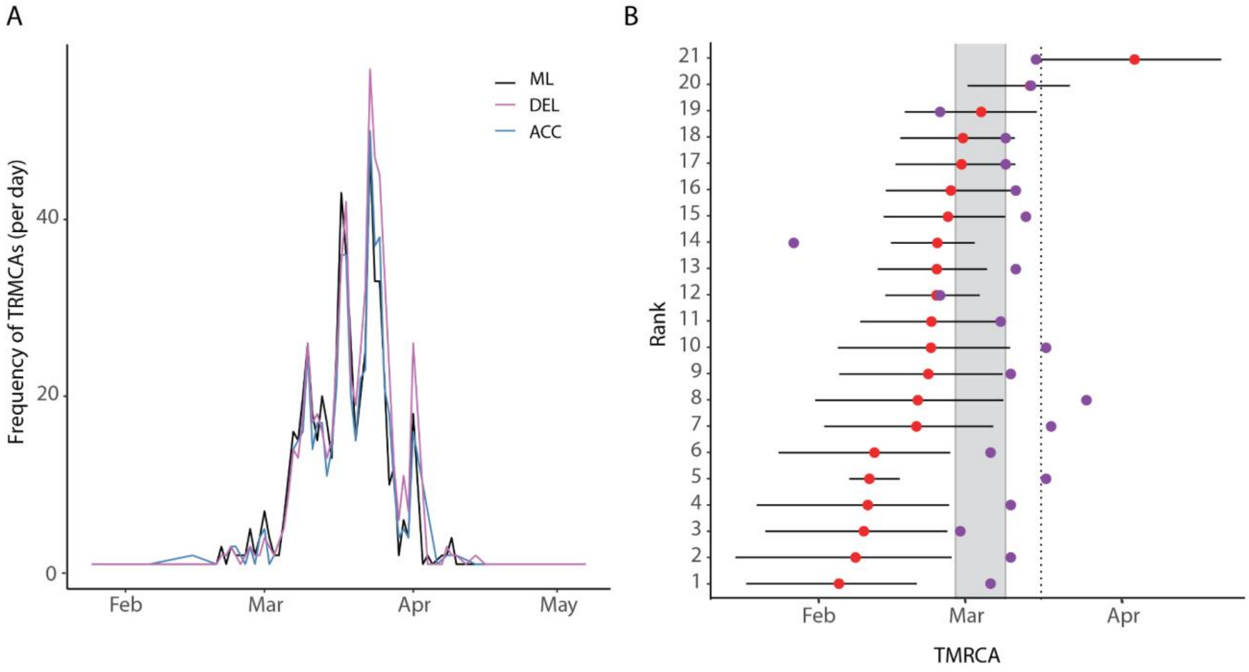

Figure S2. (A) Comparison of TMRCA point estimates inferred from the global context tree, using three ASR methods (ML, ACCtran or DELtrain) applied to the TreeTime phylogeny. (B) Rank plot of the TMRCA estimates from BEAST runs (red dots = medians, error bars = 95% HPD) of the largest transmission lineages (20 or more sequenced cases). Transmission lineages are ranked according to their median BEAST-inferred TMRCA. Purple dots are TMRCAs of the lineages but inferred using ML ASR on the TreeTime global context tree. Grey bar is the spring break period, dotted line is the day the border was closed.

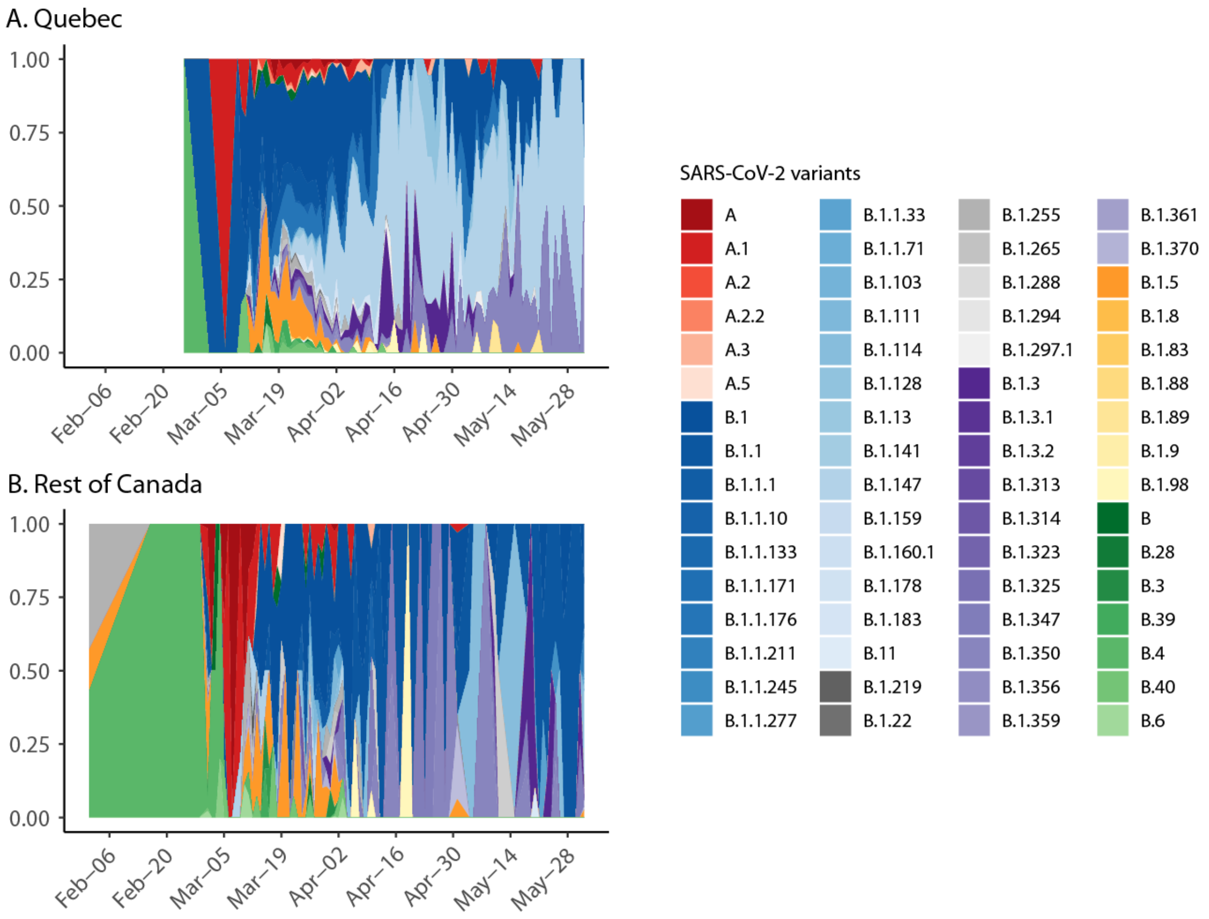

Figure S3. SARS-CoV-2 clades observed over time in (A) Québec and (B) the rest of Canada. Frequencies of named SARS-CoV-2 lineages (Pangolin nomenclature; cov-lineages.com) of sampled Québec genomes over time.

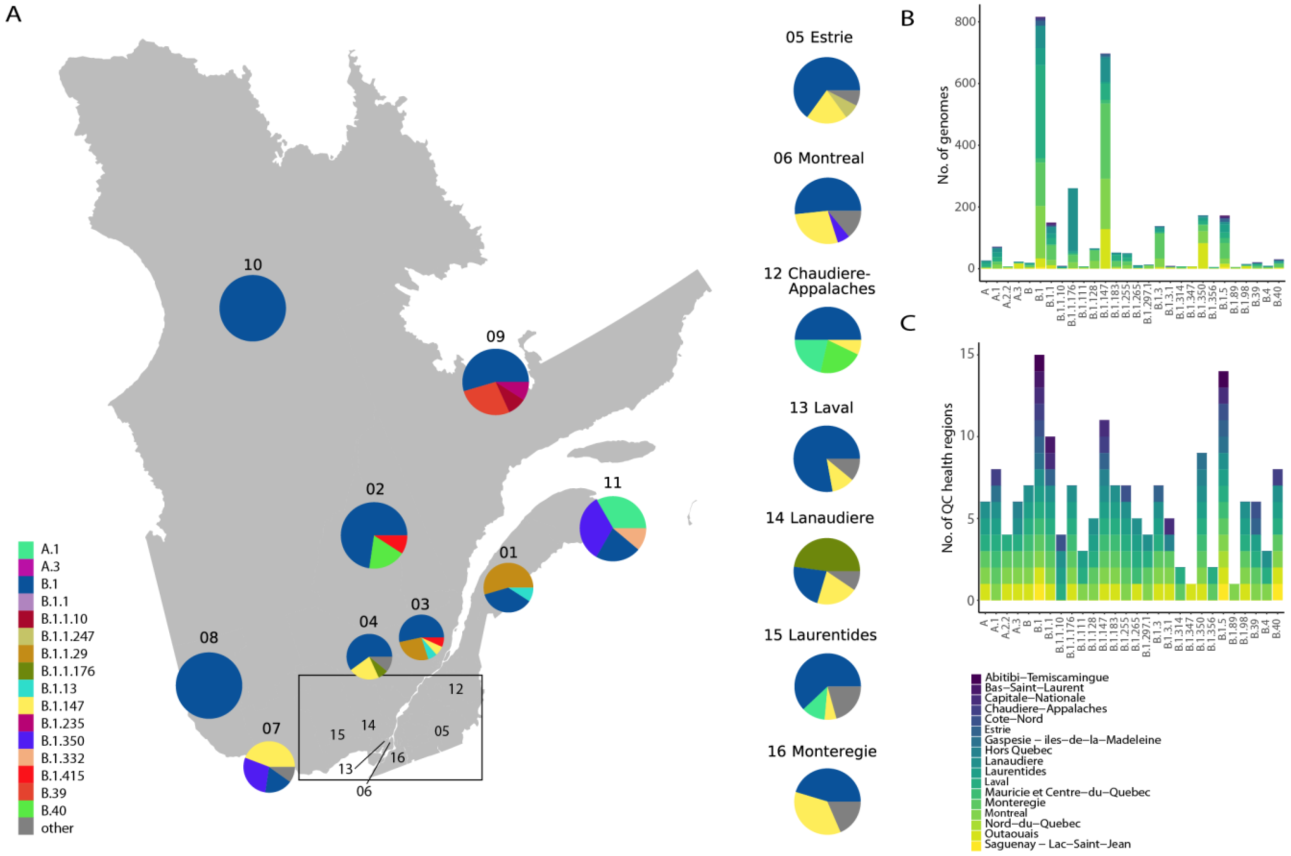

Figure S4. Geographical distribution of viral lineages in Québec. (A) Regional distribution of viral lineages, up until June 1st, 2020. (B) Frequency of viral lineages that have >3 genomes in the Québec dataset per health region. (C) Number of Québec health regions in which each named viral variant (Pangolin nomenclature) was found. Québec health regions not named in figure: 01-Bas-Saint-Laurent, 02-Saguenay-Lac-Saint-Jean, 03-Capitale-Nationale, 04-Mauricie-et-Centre-du-Québec, 07-Outaouais, 08-Abitibi-Témiscamingue, 09-Côte-Nord, 10-Nord-du-Québec, 11-Gaspésie-Iles-de-la-Madeleine. Note: Hors-Québec represents non-residents (visitors) who tested positive.

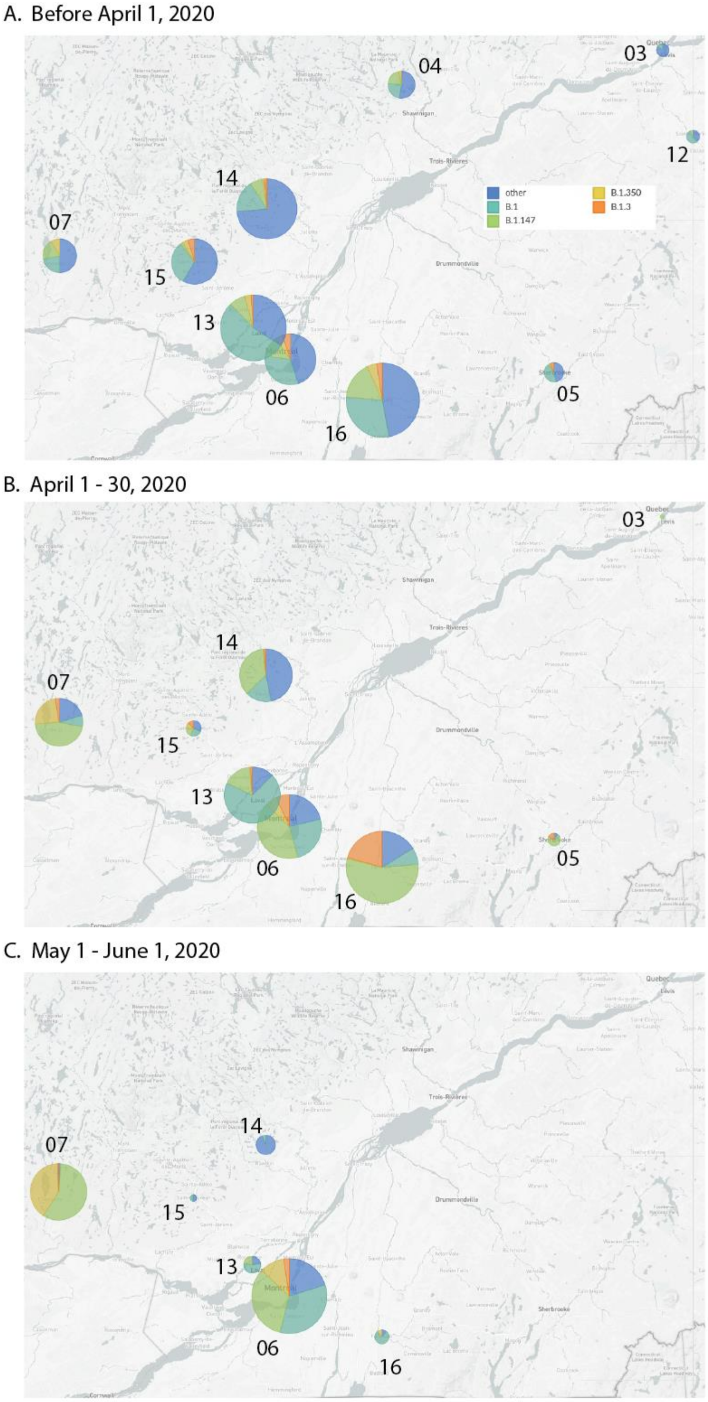

Figure S5. Temporal variation of geographical spread of the most successful viral lineages in the most populous Southern regions of Québec. Maps with pie charts showing the frequencies of viral lineages (A) up to April 1st, during exponential increase of the epidemic, (B) during the plateau of epidemic, and (C) during the decline period. Pie charts are sized proportionally to the total number of sequences sampled in the health region. The Québec health regions depicted here are: 03-Capitale-Nationale, 04-Mauricie-et-Centre-du-Québec, 05-Estrie, 06-Montréal, 07-Outaouais, 12-Chaudière-Appalaches, 13-Laval, 14-Lanaudière 15-Laurentides, 16-Montérégie

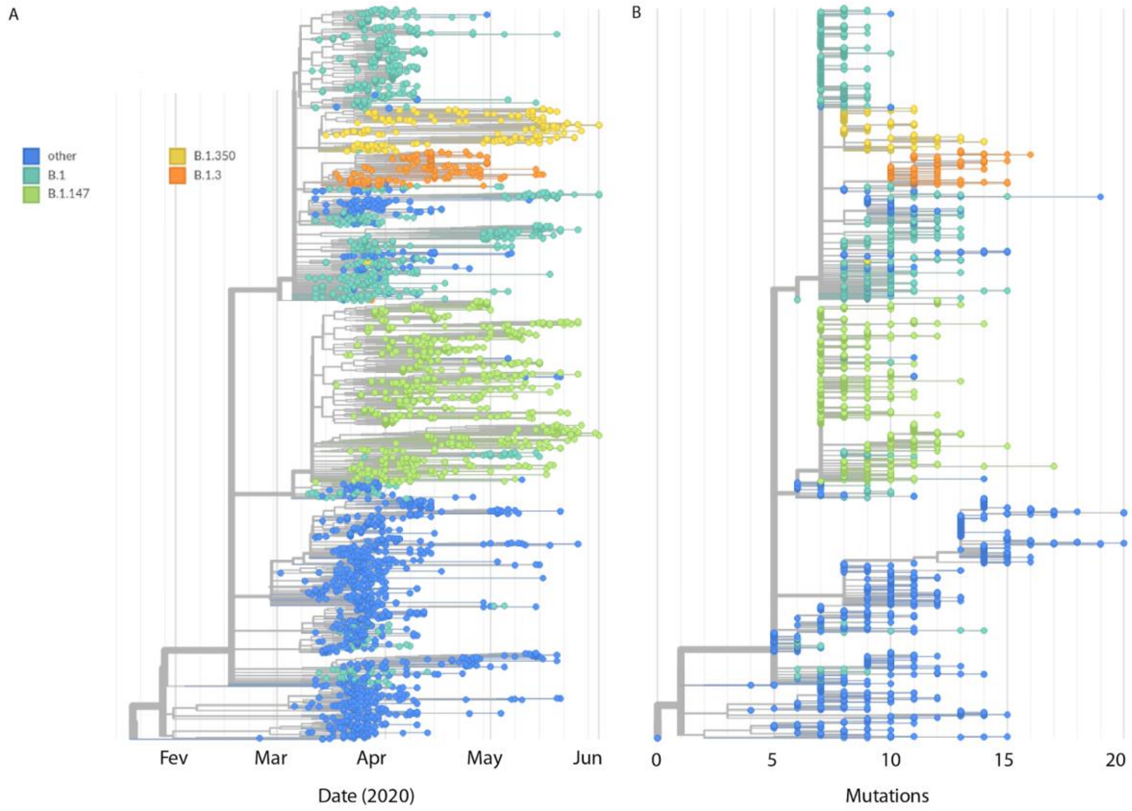

Figure S6. Phylogeny of SARS-CoV-2 genomes sampled in Québec, up to June 1st, 2020. (A) Maximum-likelihood time-scaled phylogenetic tree of all 2,921 Québec sequences from this study. The most successful viral lineages are annotated by color. (B) Divergence tree of the same dataset.

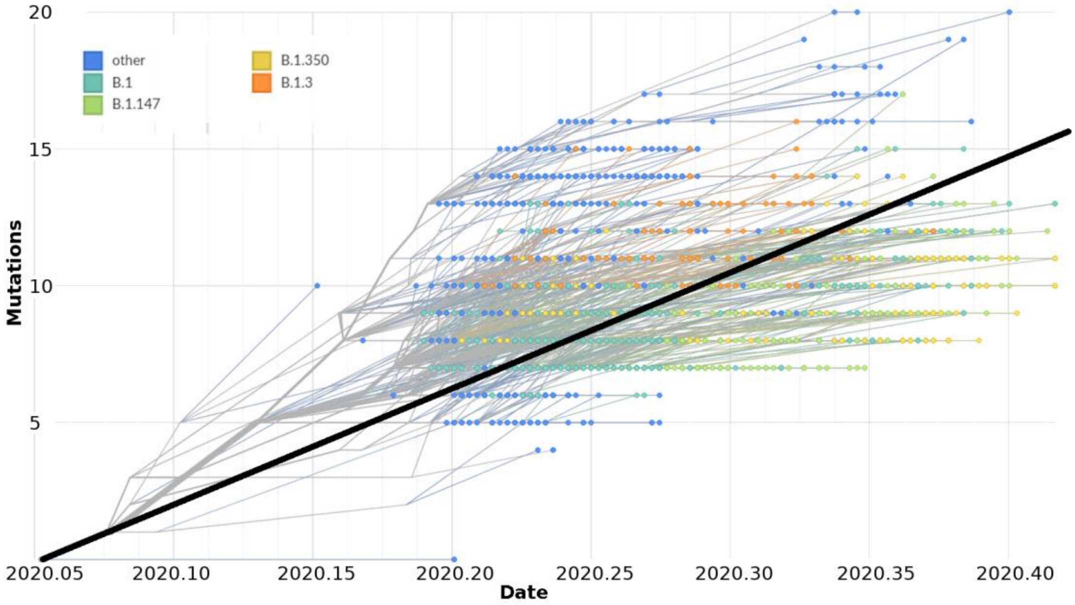

Figure S7. Root-to-tip regression analysis of Québec SARS-CoV-2 genomes up to June 1st, 2020. Most successful viral lineages are annotated by colors. This dataset exhibits a weak association between genetic distances and sampling dates (correlation coefficient, r = 0.33, R^2 = 0.11, slope = 5.65×10^{-4} substitutions/site/year, $p < 0.0001$), calculated with TempEst (Rambaut et al. 2016) using the maximum-likelihood tree displayed in Figure S4 B.

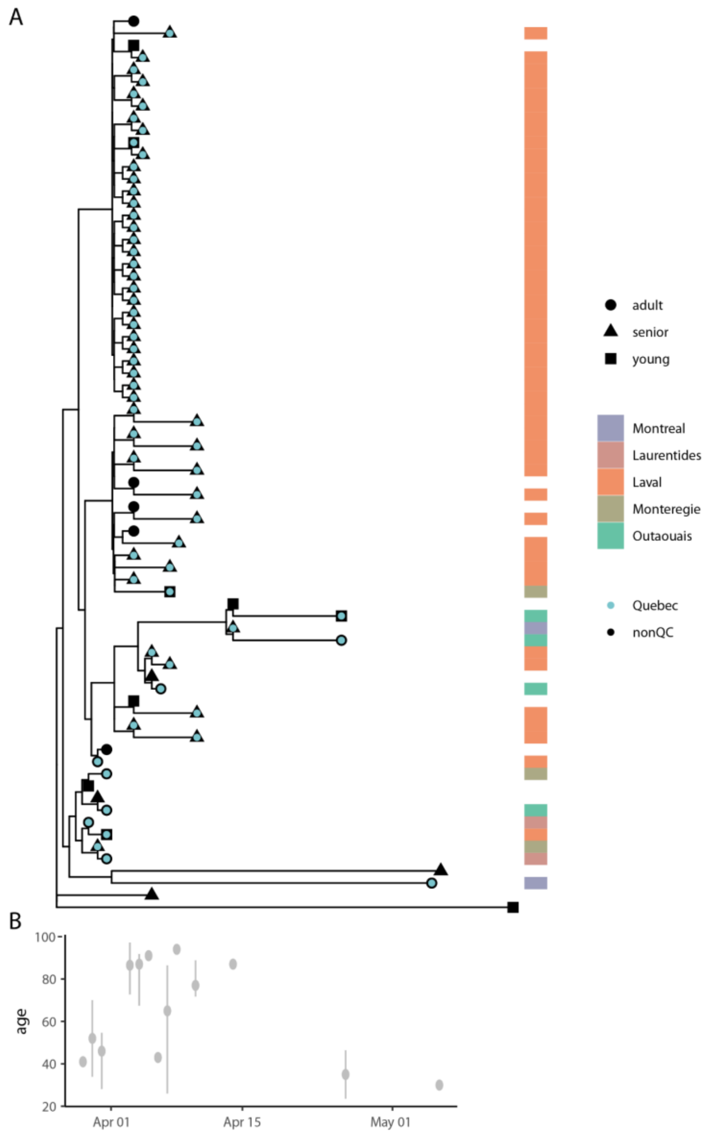

Figure S8. (A) Subtree of the time-scaled global phylogeny that captures a transmission lineage of B.1 that spread through a Laval long-term care facility. Black: consensus sequences from GISAID, not from Québec, Blue: Québec viral sequences. Young: < 30 years old, Adult: 30 - 60 years old, Senior: > 60 years old. (B) Age distribution of the Québec cases in this transmission lineage, median age (range).

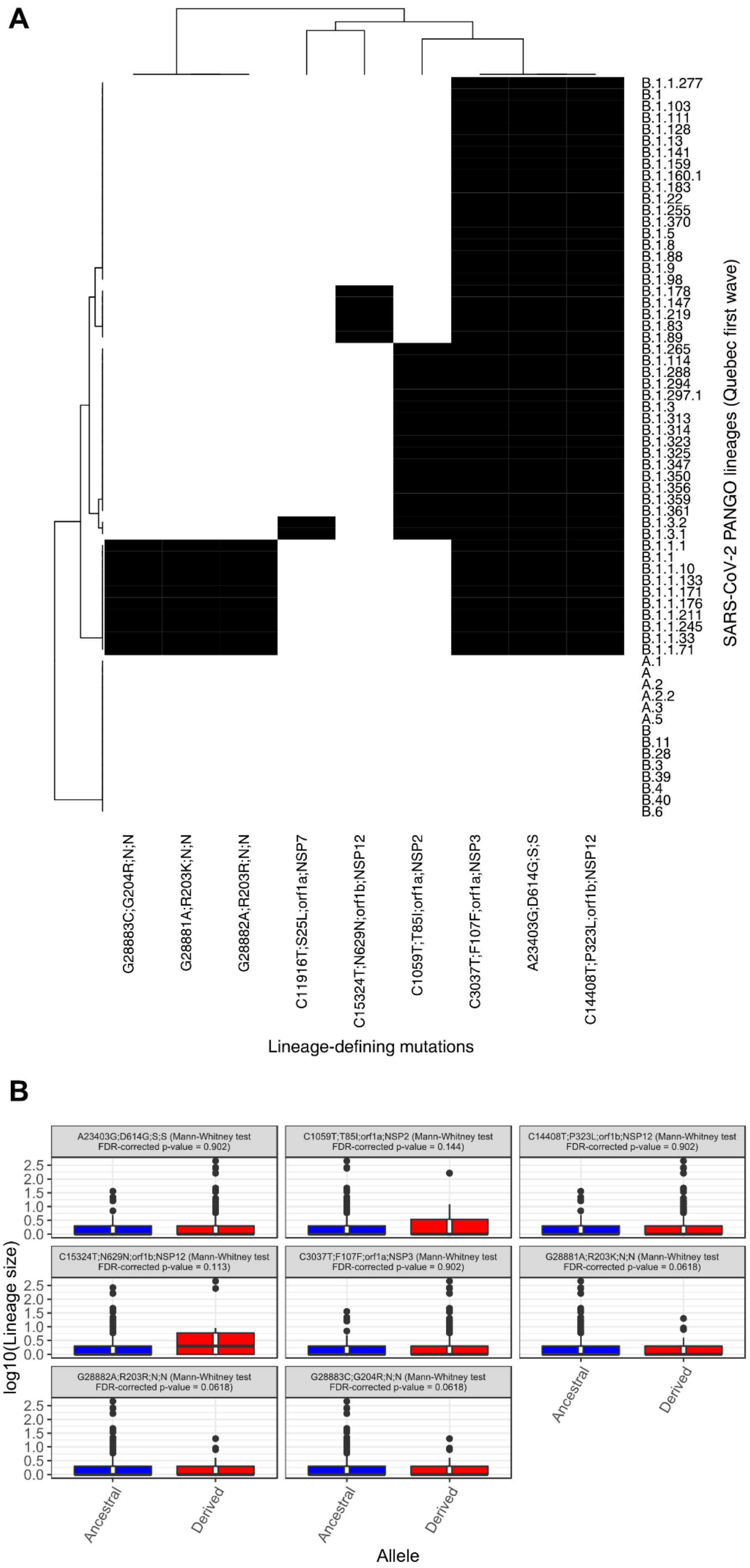
A
B.1.1.277
B.1
B.1.103
B.1.111
B.1.128
B.1.13
B.1.141
B.1.159
B.1.160.1
B.1.183
B.1.22
B.1.255
B.1.370
B.1.5
B.1.8
B.1.88
B.1.9
B.1.98
B.1.178
B.1.147
B.1.219
B.1.83
B.1.89
B.1.265
B.1.114
B.1.288
B.1.294
B.1.297.1
B.1.3
B.1.313
B.1.314
B.1.323
B.1.325
B.1.347
B.1.350
B.1.356
B.1.359
B.1.361
B.1.3.2
B.1.3.1
B.1.1.1
B.1.1
B.1.1.10
B.1.1.133
B.1.1.171
B.1.1.176
B.1.1.211
B.1.1.245
B.1.1.33
B.1.1.71
A.1
A
A.2
A.2.2
A.3
A.5
B
B.11
B.28
B.3
B.39
B.4
B.40
B.6
SARS-CoV-2 PANGO lineages (Quebec first wave)
G28883C;G204R;N;N
G28881A;R203K;N;N
G28882A;R203R;N;N
C11916T;S25L;orf1a;NSP7
C15324T;N629N;orf1b;NSP12
C1059T;T85I;orf1a;NSP2
C3037T;F107F;orf1a;NSP3
A23403G;D614G;S;S
C14408T;P323L;orf1b;NSP12
Lineage-defining mutations
B
A23403G;D614G;S;S (Mann-Whitney test FDR-corrected p-value = 0.902)
C1059T;T85I;orf1a;NSP2 (Mann-Whitney test FDR-corrected p-value = 0.144)
C14408T;P323L;orf1b;NSP12 (Mann-Whitney test FDR-corrected p-value = 0.902)
C15324T;N629N;orf1b;NSP12 (Mann-Whitney test FDR-corrected p-value = 0.113)
C3037T;F107F;orf1a;NSP3 (Mann-Whitney test FDR-corrected p-value = 0.902)
G28881A;R203K;N;N (Mann-Whitney test FDR-corrected p-value = 0.0618)
G28882A;R203R;N;N (Mann-Whitney test FDR-corrected p-value = 0.0618)
G28883C;G204R;N;N (Mann-Whitney test FDR-corrected p-value = 0.0618)
log10(Lineage size)
0.0
0.5
1.0
1.5
2.0
2.5
Ancestral
Derived
Allele

Figure S9. Associations between lineage-defining mutations and transmission lineage size. (A) Lineage-defining mutations. For each SARS-CoV-2 Pangolin viral lineage observed in Québec during the first wave, the nine mutations (columns) that are present in the consensus sequences of all the instances of the lineage (rows) are represented in black. The resulting heatmap was ordered by hierarchical clustering. (B) Associations between lineage-defining mutations and transmission lineage size. Each of the nine lineage-defining mutations was tested for association with transmission lineage size by comparing the transmission lineages size with the ancestral allele (blue) to the derived allele (red) (Mann-Whitney test, *P*-values reported after false-discovery rate correction for multiple hypothesis testing). The lineage-defining mutations are labeled in the following format: Ancestral nucleotide allele, Genome position, Derived nucleotide allele; Ancestral Amino Acid allele, Position In Protein, Derived Amino Acid allele; ORF; Gene.

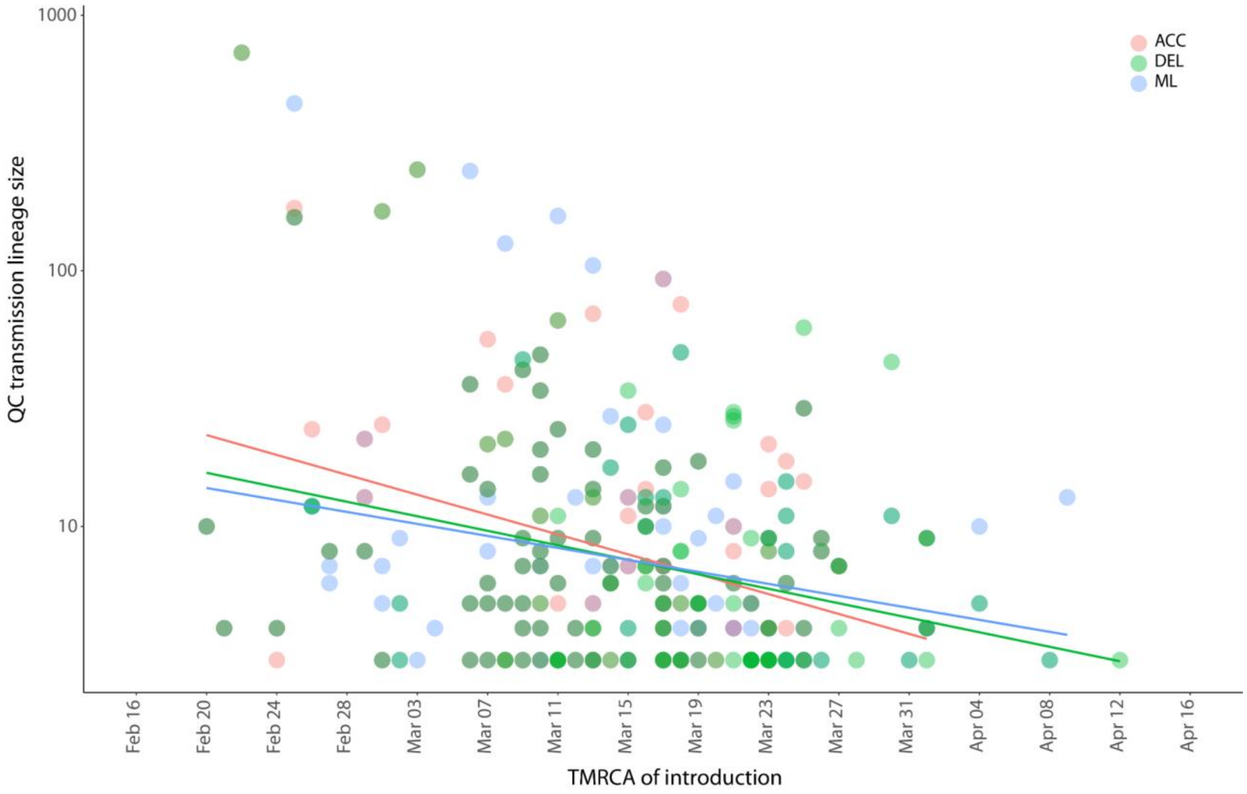

Figure S10. Relationship between transmission lineage size and their estimated TMRCA. Singletons not included. Pearson correlation coefficients, 95% CIs and p-values. ML: r = -0.30 (-0.43, -0.16), p = 6.828e-05, ACC: r = -0.39 (-0.52, -0.24), p = 1.715e-06, DEL: r = -0.32 (-0.45, -0.17), p = 3.966e-05
